# Clinical Breakpoint of Apramycin to Swine *Salmonella* and Its Effect on Ileum Flora

**DOI:** 10.3390/ijms23031424

**Published:** 2022-01-26

**Authors:** Xinyu Dai, Yufeng Gu, Jinli Guo, Lingli Huang, Guyue Cheng, Dapeng Peng, Haihong Hao

**Affiliations:** 1 National Reference Laboratory of Veterinary Drug Residues and MOA Key Laboratory for Detection of Veterinary Drug Residues, Huazhong Agricultural University, Wuhan 430070, China; 15207157890@163.com (X.D.); guyufeng@webmail.hzau.edu.cn (Y.G.); guojl@personalbio.cn (J.G.); huanglingli@mail.hzau.edu.cn (L.H.); chengguyue@mail.hzau.edu.cn (G.C.); 2 MOA Laboratory for Risk Assessment of Quality and Safety of Livestock and Poultry Products, Huazhong Agricultural University, Wuhan 430070, China

**Keywords:** *Salmonella*, apramycin, wild-type cutoff value, PK/PD cutoff value, clinical cutoff value, clinical breakpoint, *16S rRNA* gene sequencing

## Abstract

The purpose of this study was to establish the clinical breakpoint (CBP) of apramycin (APR) against *Salmonella* in swine and evaluate its effect on intestinal microbiota. The CBP was established based on three cutoff values of wild-type cutoff value (CO_WT_), pharmacokinetic-pharmadynamic (PK/PD) cutoff value (CO_PD_) and clinical cutoff value (CO_CL_). The effect of the optimized dose regimen based on ex vivo PK/PD study. The evolution of the ileum flora was determined by the *16rRNA* gene sequencing and bioinformatics. This study firstly established the CO_WT_, CO_PD_ in ileum, and CO_CL_ of APR against swine *Salmonella*, the value of these cutoffs were 32 µg/mL, 32 µg/mL and 8 µg/mL, respectively. According to the guiding principle of the Clinical Laboratory Standards Institute (CLSI), the final CBP in ileum was 32 µg/mL. Our results revealed the main evolution route in the composition of ileum microbiota of diarrheic piglets treated by APR. The change of the abundances of *Bacteroidetes* and *Euryarchaeota* was the most obvious during the evolution process. *Methanobrevibacter*, *Prevotella*, *S24-7* and *Ruminococcaceae* were obtained as the highest abundance genus. The abundance of *Methanobrevibacter* increased significantly when APR treatment carried and decreased in cure and withdrawal period groups. The abundance of *Prevotella* in the tested groups was significantly lower than that in the healthy group. A decreased of abundance in *S24-7* was observed after *Salmonella* infection and increased slightly after cure. *Ruminococcaceae* increased significantly after *Salmonella* infection and decreased significantly after APR treatment. In addition, the genera of *Methanobrevibacter* and *Prevotella* were defined as the key node. Valine, leucine and isoleucine biosynthesis, D-Glutamine and D-glutamate metabolism, D-Alanine metabolism, Peptidoglycan and amino acids biosynthesis were the top five Kyoto Encyclopedia of Genes and Genomes (KEGG) pathways in the ileum microbiota of piglets during the *Salmonella* infection and APR treatment process. Our study extended the understanding of dynamic shift of gut microbes during diarrheic piglets treated by APR.

## 1. Introduction

Salmonellosis is one of the most frequent foodborne zoonotic diseases, characterised by abdominal cramps, diarrhoea, fever and vomiting, constituting a worldwide major public health concern [1]. Breeding sows asymptomatically infected with *Salmonella* can be naturally transmitted directly or indirectly between animals and humans, for example through the consumption of contaminated animal derived food or through contact with infected animals [2]. The aminoglycoside antibiotics interfered with translational fidelity by binding to the 30S ribosomal subunit, exhibiting excellent characteristics as broad-spectrum antibacterial agents with desirable bactericidal activity against Gram-negative bacteria and mycobacteria [3,4]. Apramycin (APR), equipping a bicyclic sugar moiety with a mono-substituted deoxystreptamine, is distinct from other aminoglycosides [5]. This distinct structure protects APR from degradation by aminoglycoside modifying enzymes that confer resistance to other aminoglycosides and contributes to offer higher selectivity for bacteria [6]. As a veterinary aminocyclitol aminoglycoside, APR was often used to treat or prevent infections caused by Gram-negative bacteria such as Colibacillosis, Salmonellosis and Enteritis in farm animals [7,8].

Epidemiological investigations of APR-resistant bacteria from foodborne animals demonstrated that prevalence of APR resistance is different among animals [9]. Cross resistance was also observed between APR and other antibiotics such as gentamicin [10,11]. However, there is no clinical breakpoint (CBP) to interpret APR against swine *Salmonella* making it impossible to interpret the results of an antimicrobial susceptibility testing (AST). CBPs are minimum inhibitory concentration (MIC) cutoff values used by diagnostic laboratories to distinguish bacteria as susceptible (S), intermediate (I) or resistant (R) to an antimicrobial agent [12]. The CBP of an antibiotic is established according to defined rules, including the wild-type cutoff values (CO_WT_) of the organism against the antimicrobial, pharmacokinetic-pharmacodynamics (PK/PD) considerations through the PK/PD cutoff values (CO_PD_), and clinical cutoff values (CO_CL_) based on clinical efficacy data [12,13]. Comprehensive procedures on setting CBPs for veterinary medicine have been established and documented in the Clinical Laboratory Standards Institute (CLSI)/Veterinary Antimicrobial Susceptibility Testing (VAST) profiles [14]. 

A highly diversified community of microorganisms parasitize in the mammalian gastrointestinal tract with approximately 10^12^ microbes containing about 500–1000 species [15]. The gastrointestinal microbiome is an enormous and dynamic ecosystem, which have a profound impact on health and disease participating in the absorption of nutrients and the expulsion of metabolic wastes from the host and shaping a barrier against the pathogens [16]. For swine, microbiota also contributes to the development of the gastrointestinal immune system and plays a considerable role in diarrhea [17]. Previous study has utilized high-throughput sequencing of the *16S rRNA* gene to characterize gut microbiota of diarrheic piglets [18]. However, gut microbiota evolution track and their correlation to treatment of piglet diarrhea with APR being poorly understood. 

The aim of the current investigation was to decide the CO_WT_, CO_PD_ and CO_CL_ values for the establishment of the CBP and to evaluate the clinical efficacy of APR against swine *Salmonella*. Then the intestinal flora changes in the composition and evolution of the piglets in healthy phase, *Salmonella* challenge phase, APR treatment phase, APR cure phase and withdrawal phase was compared and analyzed to identify hub differences in the gastrointestinal tract microbiota of piglets to reveal diarrhea-related bacteria.

## 2. Results

### 2.1. MIC Distribution and CO_WT_

**Figure 1:**
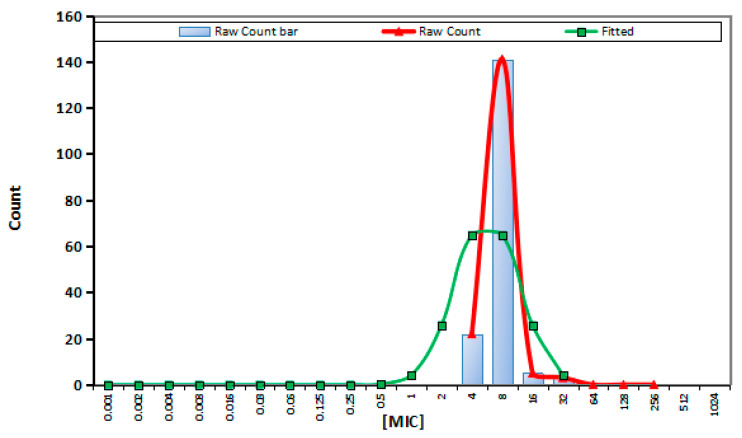
Nonlinear regression of MIC distribution for APR against swine *Salmonella*.

MICs of APR against 185 swine *Salmonella* isolates were shown in Table S1. The MICs was distributed in a normal distribution pattern with a definite peak at 8 μg/mL. The corresponding MIC_50_ and MIC_90_ were determined to be 8 and 16 μg/mL, respectively. The fitted MIC distribution, encompassing 95% of the wild-type isolates, was processed in Ecoffinder software and the epidemiological cutoff (ECOFF) value was therefore calculated to be 32 μg/mL (Figure 1). 

### 2.2. PD Study of APR against Swine Salmonella Strain 10# In Vitro and Ex Vivo

**Figure 2:**
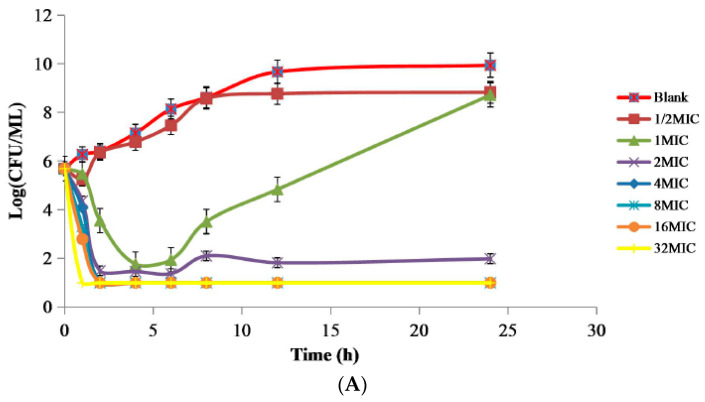

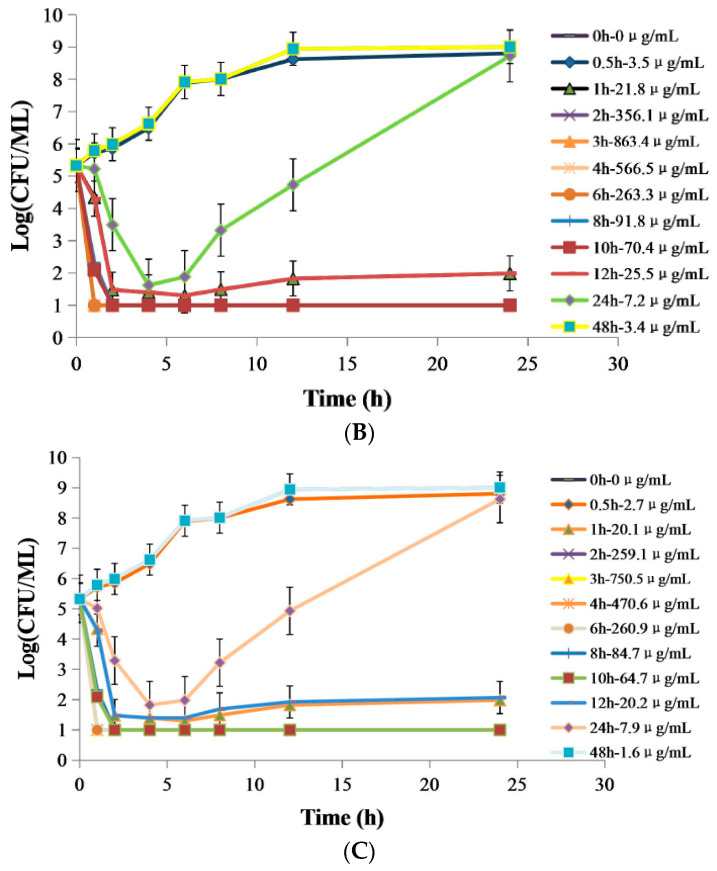
The time–killing curve of APR against swine *Salmonella* in vitro and ex vivo. (**A**) The time-killing curve in MH broth; (**B**) the time–killing curve in ileum fluid of health piglets; (**C**) the time–killing curve in ileum fluid of diarrhea piglets.

The MIC and minimum bactericidal concentration (MBC) values of APR against swine *Salmonella* 10# were 8 and 16 μg/mL both in Mueller-Hinton (MH) broth and ileum fluid. The mutant prevention concentration (MPC) of APR against swine *Salmonella* 10# was 51.2 μg/mL. The Post-antibiotic effect (PAE) of swine *Salmonella* exposed to APR for 1 h and 2 h were ranging from 0.51–1.42 h, 0.45–6 h, respectively (Table S2). The in vitro time-killing curves showed that APR displayed a concentration-dependent bactericidal effect as increasing drug concentrations induced more rapid and radical killing effects (Figure 2A). Sustained growth inhibition was observed only within the initial 6 h in the MH broth containing lower than 2 × MIC concentration of APR. When 10# was exposed to APR at a concentration equal to or higher than 2 × MIC, the total bacteria were significantly decreased to the undetectable level (≤10 CFU/mL) after 24 h of APR exposure. As presented in Figure 2B,C, the bacteria were drastically reduced to the undetectable limit (<10 CFU/mL) after exposure to the ileum fluid collected between 2–10 h after oral administration, suggesting that APR exhibited a concentration-dependent killing mechanism in the ex vivo environment, consistent with the in vitro bactericidal effect. Slight regrowth was observed in the ileum fluid samples collected at 1 and 12 h after incubation for 6 h. 

### 2.3. PK/PD Analysis in the Plasma and Ileum Fluid and Establishment of CO_PD_

**Figure 3:**
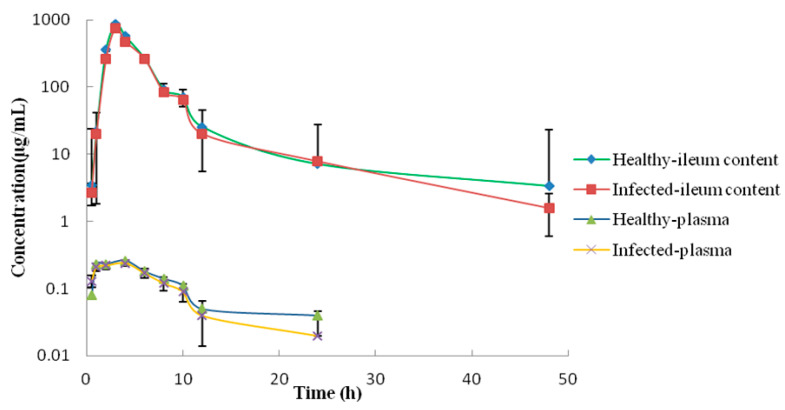
Concentration–time curve of APR in plasma and ileum content of piglets.

Single-dose PK of APR in plasma and ileum contents was shown in Figure 3. Concentrations of APR in plasma and ileum contents at a series of time points were shown in Tables S3 and S4. Remarkable gap of drug concentrations was observed between plasma and ileum. The absorption of APR after oral administration was limited, but the concentration in the ileum fluid was markedly higher compared to the APR concentration in plasma (Table 1). The AUC_0–24h_ value in ileal contents was also significantly higher than that in plasma. The C_max_ in plasma was far lower than the MIC of the selected strain 10#, so the APR could not achieve the purpose of treatment. Therefore, the PK parameters of ileal contents were selected for PK/PD analysis.

The relationship between the antimicrobial efficacy and the ex vivo PK/PD parameter of AUC_0–24h_/MIC ratios was fitted by using the inhibitory sigmoid E_max_ model. The model parameters, including the Hill coefficient (N), E_0_, E_max_ and AUC_0–24h_/MIC values for the three levels of growth inhibition were presented in Table 2; the AUC_0–24h_/MIC values for bacteriostatic activity (E = 0), bactericidal activity (E = −3) and a virtual elimination effect (E = −4) were 46.65, 48.90 and 49.98 h in healthy piglets, respectively. The AUC_0–24h_/MIC values for bacteriostatic activity (E = 0), bactericidal activity (E = −3), and a virtual elimination effect (E = −4) were 46, 58.38 and 65.53 h in infection piglets, respectively. Based on Monte Carlo Simulation (MCS), the probability of target attainment (PTA) under different MICs was calculated (Table S5). The maximum MIC with a PTA equal to or greater than 90% was selected as CO_PD_. Therefore, the CO_PD_ of APR against *Salmonella* was 32 μg/mL.

### 2.4. Dosage Determination and Establishment of CO_CL_ of APR against Swine Salmonella 

According to the MCS, the ratio of CL/f and AUC_0–24h_/MIC under different antibacterial effects were obtained. The doses predicted to obtain bacteriostatic, bactericidal and elimination effects for *Salmonella* over 24 h were 4.15, 5.27, 5.91 mg/kg b.w. Statistics of clinical trial results were shown in Table S6. Then the results of the obtained clinical MIC and Probablity of Cure (POC) were analyzed by the “WindoW” method, and the selection threshold of the CO_CL_ was 8–32 μg/mL. A CO_CL_ value obtained by Correlation and Regression Trees (CART) analysis was to be lower than 12 μg/mL. Combining the above two analytical methods, the CO_CL_ value was calculated as 8 μg/mL.

### 2.5. DNA Sequence Data and Bacterial Community Structure 

**Figure 4:**
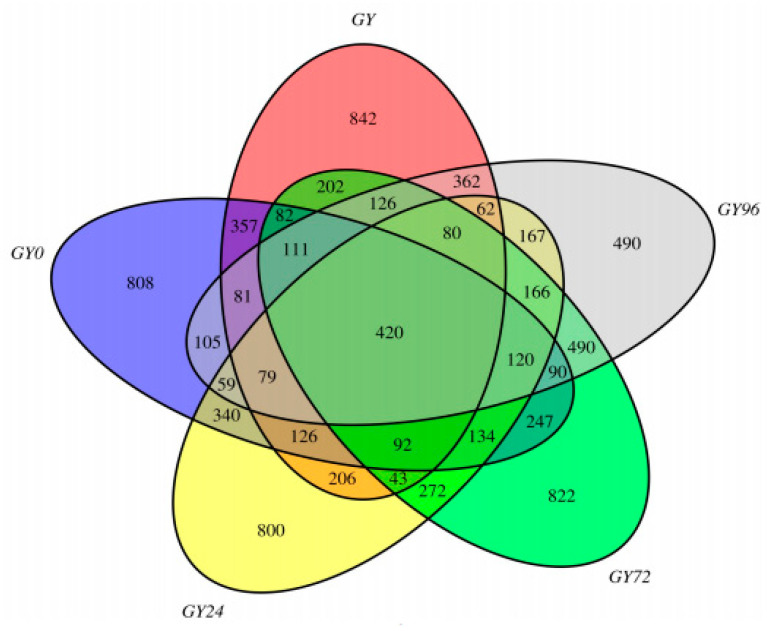
The Venn diagram of the shared and unique OTUs presented in different groups. Note: “GY” represents the healthy group, “GY0” represents the infection group, “GY24” represents the treatment group, “GY72” represents the cure group and “GY96” represents the withdrawal period group.

Based on 97% species similarity, all the sequences were clustered into operational taxonomic units (OTUs) with the size ranging from 1570 to 2638 (Table S7). The rarefaction curves (Figure S1) showed that the sequencing depth was near saturation and that the sequencing data included most of the *16S rRNA* gene information. A total of 8381 OTUs were identified from all ileum content samples, with 420 of those existing in each group defined as core OTUs (Figure 4). The core OTUs comprised approximately 5% of the total OTUs while 842, 808, 800, 822 and 490 OTUs were uniquely identified in the healthy, infection, treatment, cure and withdrawal period group, respectively.

**Figure 5:**
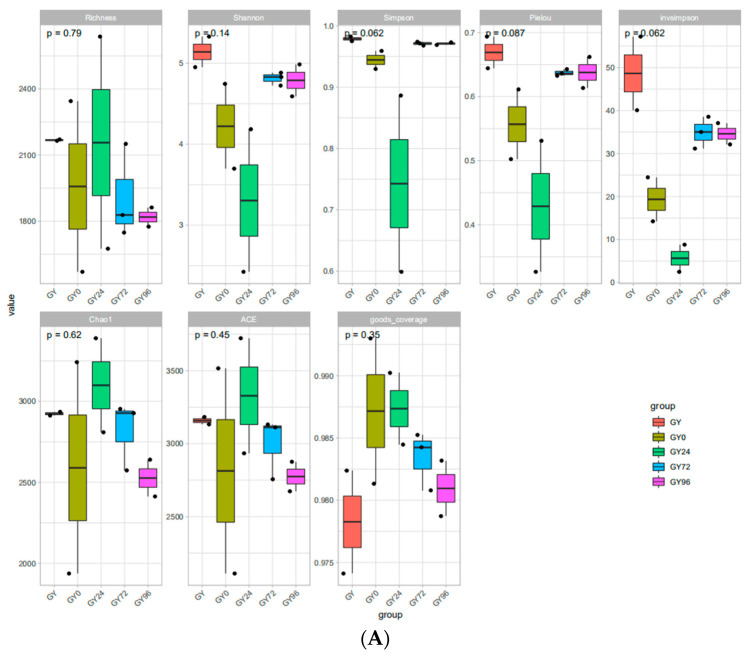

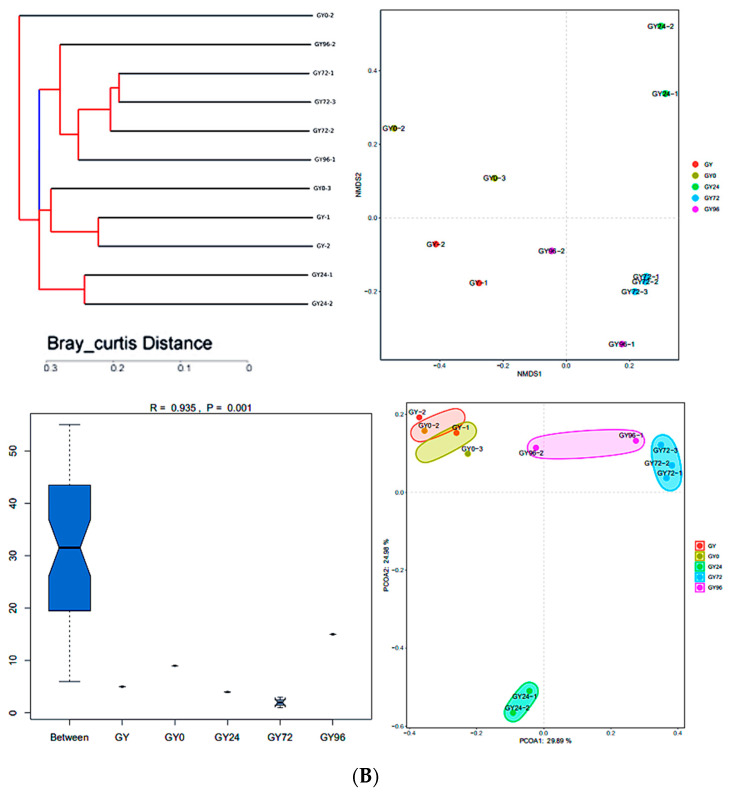
Alpha and beta diversity parameters presented in the five groups. (**A**) Alpha diversity parameters of richness, Shannon, Simpson, pielou, invsimpson, Chao1, ACE and good coverage; (**B**) Beta parameters of UPGMA, NMDS, ANOSIM and PCoA.

The microbial complexities in the healthy, infection, treatment, cure and withdraw period groups were demonstrated by Alpha diversity parameters, including richness, Shannon, Simpson, pielou, invsimpson, Chao1, Abundance-based Coverage Estimator (ACE) and good coverage (Figure 5A). The results showed that healthy group samples had the largest alpha-diversity indices followed by those in the cure group, withdrawal period group, infection group and treatment group. To evaluate overall differences in beta-diversity, the unweighted pair group method with arithmetic mean clustering (UPGMA), nonmetric multidimensional scaling (NMDS) analysis, the ANOSIM and the principal coordinate analysis (PCoA) was used to identify discrepancies between groups. As shown in Figure 5B, the results of UPGMA and NMDS analysis indicated the clear separation of the bacterial community structure into mainly two large clusters. This difference between the bacterial communities was significant, as determined using the ANOSIM analysis (R = 0.935, *p* = 0.001). PCoA results showed that the ileum microbiota of the healthy group was similar to that of the infection group, and the ileum microbiota of the cure group was similar to that of the withdrawal period. Specifically, the ileum microbiota of the treatment group was distinct to other groups. Collectively, these data point toward the marked difference in the ileum microbiota between these groups.

### 2.6. Analysis of Microbial Flora Composition in Ileum of Piglets under Different Taxonomic Levels

**Figure 6:**
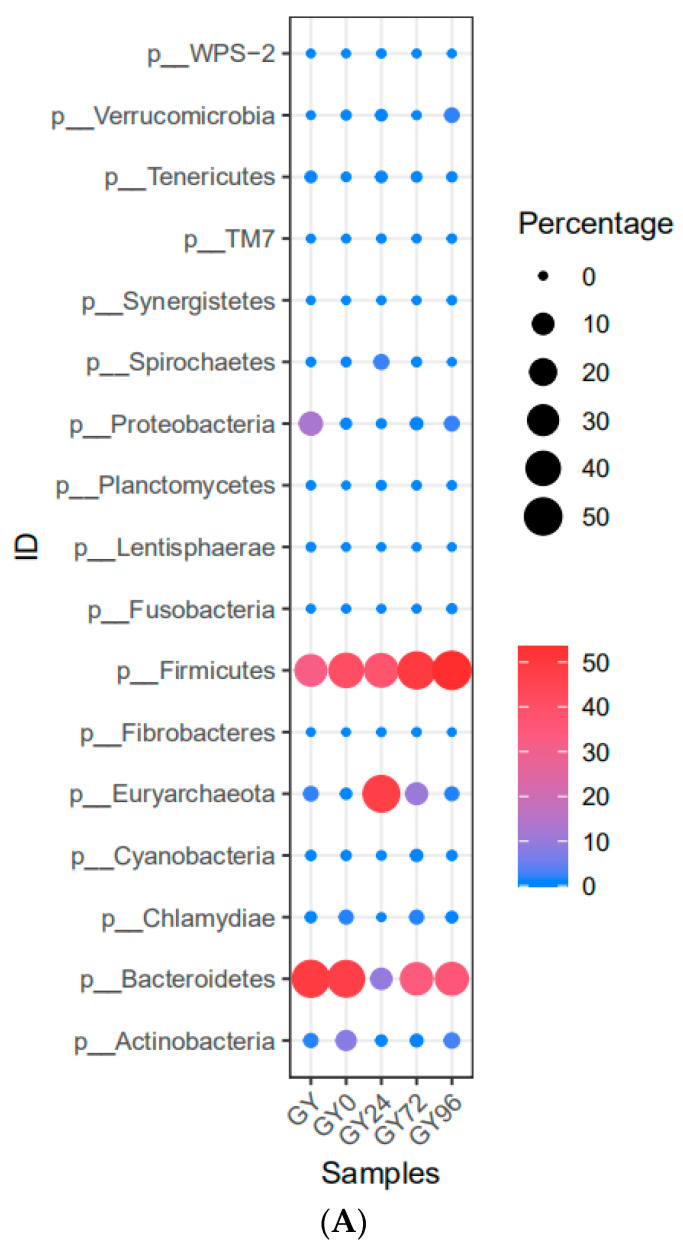

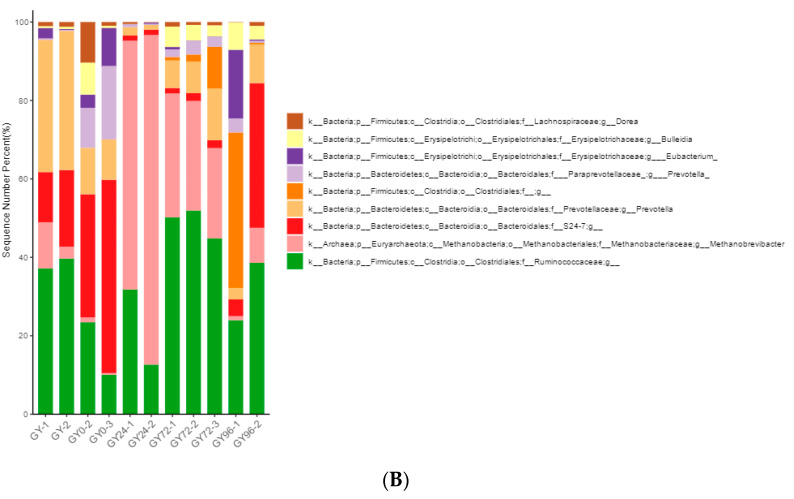
Taxonomic classification of the *16S rRNA* gene sequences at phylum and genus levels. (**A**) The evolution Flora presented at phylum level; (**B**) The bacterial community composition at genus level.

OTUs obtained from samples belonged to 17 phyla, 33 classes, 50 orders, 95 families and 177 genera. Notably, at the phylum level, *Firmicutes* was the most predominant phylum ranging from 32.23% to 53.53%. The abundances of *Firmicutes* were relatively small increase form *Salmonella* infection to withdrawal period. The *Euryarchaeota* was significantly increased in the treatment group (from 2.25% to 47.62%) and quickly decreased to the initial level. The shifts of the abundances of *Bacteroidetes* were precisely opposite to that of the *Euryarchaeota* and ultimately return to the initial state (Figure 6A). There was also a conspicuous abundances change in the *Actinobacteria* and *Proteobacteria*, but no significant differences in other phylum were discerned in these groups. In general, the use of APR in the treatment of piglet diarrhea would not destroy the balance of intestinal flora at the phylum level. Evidently, *Prevotellaceae* belonging to the phylum *Bacteroidetes* and *Ruminococcace*, belonging to the phylum *Firmicutes*, were the top two abundant families in the ileum. The family *S24-7* had significantly lower abundance than *Prevotellaceae* and *Ruminococcace* in the healthy group (Figure 6B).

At the genus level, the top four most abundant genera containing 63.25–98.95% of the total OUTs were *Methanobrevibacter*, *Prevotella*, *S24-7* and *Ruminococcaceae*. *Methanobrevibacter* suddenly increased significantly from APR treatment and decreased slightly in the cure group. The percentage of *Prevotella* in all other groups was significantly lower than that in the healthy group. A decreased of S24-7 was observed after *Salmonella* infection and increased slightly after cure. *Ruminococcaceae* increased significantly after *Salmonella* infection and decreased significantly after APR treatment (Figure 6B).

**Figure 7:**
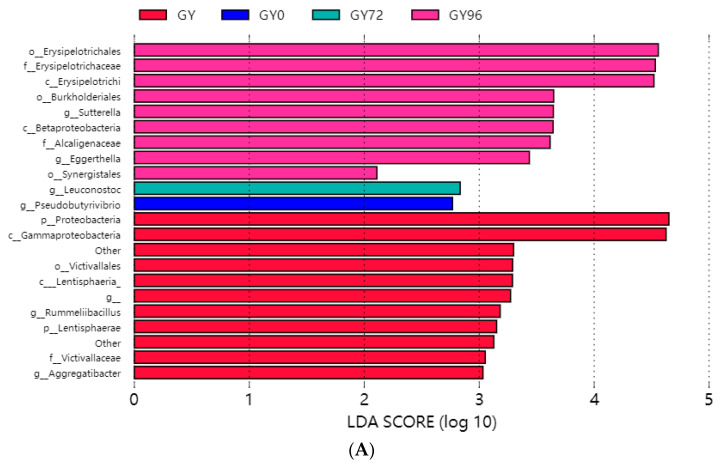

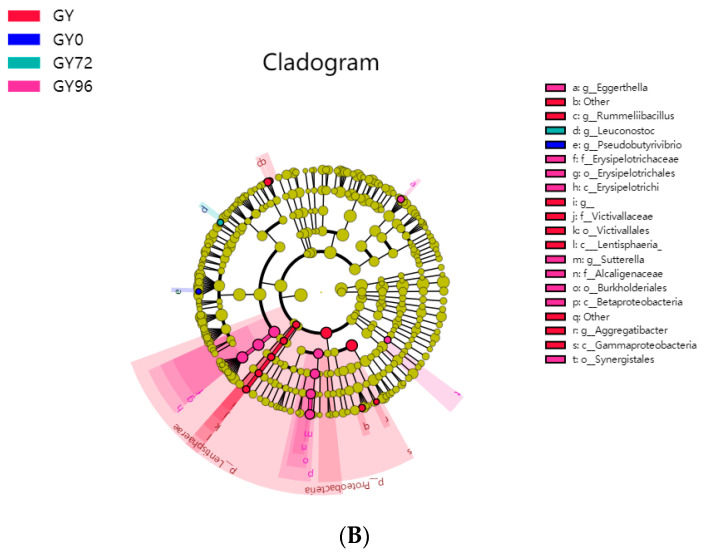
Different structures of ileum microbiota presented in different groups by the LEfSe analysis. (**A**) Taxonomic biomarkers found among groups by LEfSe; (**B**) Cladogram plot of the biomarkers. The size of the node represents the abundance of the taxa. Only taxa with LDA scores (log 10) > 4 were shown.

Linear Discriminant Analysis Effect Size (LEfSe) analysis was used to identify biomarkers of ileum microbiota of different stages of clinical trial piglets (Figure 7). The differentially abundant phyla detected showed that the *Proteobacteria* and *Lentisphaerae* phylum was predominant in the healthy group, while the most abundant genus in the infection group was *Pseudobutyrivibrio*. It was found that the *Leuconostoc* was a predominant genus in the cure group, whereas the *Eggerthella*, *Leuconostoc* and *Sutterella* were found to be a dominant genus in withdraw period (Figure 7A). There was an overrepresentation of *Proteobacteria* and *Lentisphaerae*, and underrepresentation of other phyla (Figure 7B).

### 2.7. Correlations of the Different Dominant Bacterial Communities 

**Figure 8:**
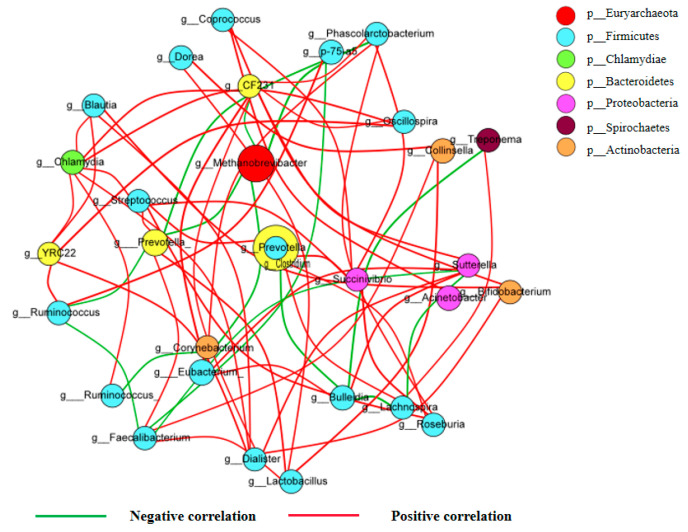
Co-occurrence network existed of ileum flora.

Through Spearman network analysis, the top 30 OTUs with the greatest absolute abundances among all samples were selected for correlation analysis at the genus level. The calculated results were plotted after filtering out differences with a *p* value greater than 0.05 or a correlation coefficient R < 0.4 (Figure 8). The top 30 genera were classified into seven phyla, including *Actinobacteria*, *Bacteroidetes*, *Chlamydiae*, *Euryarchaeota*, *Firmicutes*, *Proteobacteria* and *Spirochaetes*. Interestingly, two genera were found as the core node, including *Methanobrevibacter* and *Prevotella*, showed highly correlation to other genus. Hence, the genera *Methanobrevibacter* and *Prevotella* might play a vital role in *Salmonella* infection and APR treatment process.

### 2.8. Predicted Kyoto Encyclopedia of Genes and Genomes (KEGG) Pathway 

**Figure 9:**
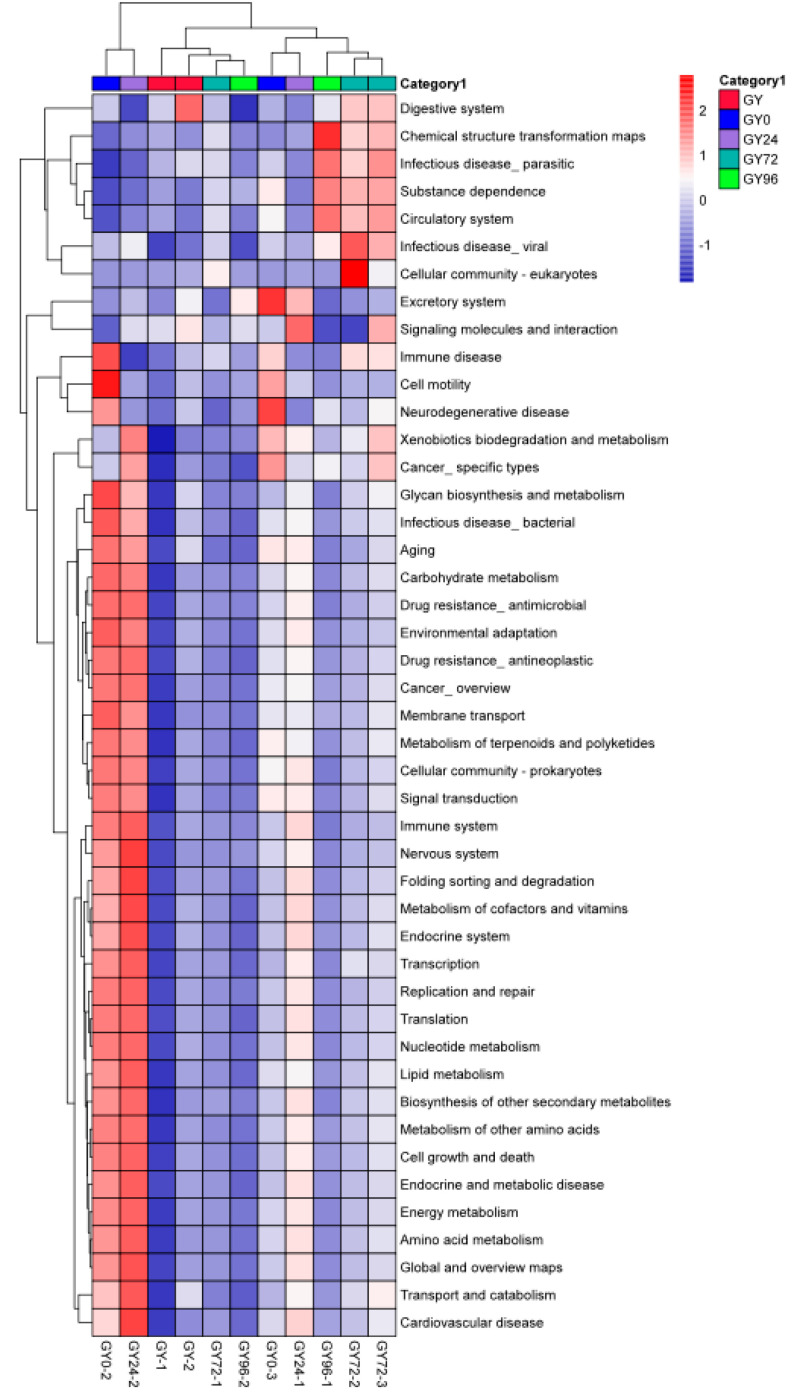
Comparison of variations presented in abundance of known KEGG pathways.

It was found that KEGG pathways involved in cellular processes, environmental information processing, genetic information processing, human diseases, metabolism and organismal systems were predominant in these groups (Figure 9). Overall, 45 pathways were obtained at level 2, and amino acid metabolism, carbohydrate metabolism, metabolism of cofactors and vitamins, metabolism of other amino acids, biosynthesis of other secondary metabolites, global and overview maps, replication and repair, glycan biosynthesis and metabolism, lipid metabolism and energy metabolism were the top ten KEGG pathways in the ileum microbiota of piglets in this study (Table S8). Moreover, 314 KEGG pathways were found at level 3. Valine, leucine and isoleucine biosynthesis, D-Glutamine and D-glutamate metabolism, D-Alanine metabolism, peptidoglycan biosynthesis and biosynthesis of amino acids were the top five KEGG pathways in the ileum microbiota of piglets.

## 3. Discussion

APR is currently used as an orally dosed, non-absorbed veterinary antibiotic to treat diarrheal diseases in poultry and livestock [19]. For APR, the American National Antimicrobial Resistance Monitoring System for animal isolates classed *Escherichia coli* (*E. coli*) and *Salmonella* as resistant if the MICs were higher than 16 μg/mL. The MICs were determined for APR of *Salmonella* (*n* = 52) isolates and the MIC_50_ and MIC_90_ values for APR were 4 and 8 μg/mL, respectively [20]. The CO_WT_ for APR of *E. coli* isolated from chicken intestinal tracts was defined as 16 to 32 μg/mL established by the National Antibiotic Resistance Monitoring Study. It was also reported that the MIC_50_ and MIC_90_ for APR against *E. coli* were 8 and 16 μg/mL, and the CO_WT_ for APR of *E. coli* was 16 μg/mL [21]. In this study, the results showed that the MIC_50_ and MIC_90_ values for APR against 185 swine *Salmonella* isolates obtained as 8 and 16 mg/L, and the CO_WT_ value for APR of *Salmonella* was 32 μg/mL. Our result was similar to the previous study suggesting that the reliability of our result was reasonably high. 

For PK/PD evaluation, tissue distribution should be considered, but the tissue distribution was often overlooked when developing PK/PD targets [22,23]. APR following oral administration with a poor absorption making most of the drugs remained in the gastrointestinal tract, which caused a great difference in the distribution of APR in plasma and intestine. PK studies on APR were mainly based on PK data in plasma. Previous result showed that the peak plasma concentration of APR was 0.23 μg/mL and reached at 2.25 h, the AUC_INF_ was 1.59 h·μg/mL in plasma after oral administration of 4 mg/kg [24]. The corresponding PK parameters of the C_max_ and AUC_INF_ were lower observed in plasma of pigs and in broiler chickens [25,26]. In our study, PK parameters were obtained both in plasma and ileum. The AUC_INF_ and C_max_ value in ileal contents were 2855.83 ± 19.81 h·μg/mL and 750.5 ± 24.82 μg/mL, respectively. The AUC_INF_ and C_max_ value in plasma were 3.41 ± 0.48 h·μg/mL and 0.24 ± 0.01 μg/mL, respectively. The result indicated that the AUC_INF_ and C_max_ in ileum were significantly higher than that in plasma. Our results were consistent with previous study that APR displayed a much lower bioavailability and was beneficial for the treatment of gastrointestinal infections. Our result was also proved that the PK parameter in ileum was more accurate for PK/PD model of intestinal infection in pigs.

At present, the dosage of APR for the treatment of Salmonellosis has not been formulated. Clinical treatment always refers to the clinical recommended dose scheme of APR in the Chinese veterinary drug code. Wild type MIC distributions and AUC_0–24h_/MIC ratios have a great effect on doses prediction. It is based on MIC_90_ for each pathogen and average values for other variables, only providing an initial indication of likely effective dosage, but does not take account either of variability or incidence of each input variable [27]. In our study, the MIC for 141 strains of total 185 strains was 8 μg/mL, so whether the MIC_90_ value can represent the actual clinical data remains to be further studied. In future studies, these concerns could be addressed by increasing numbers of isolates in field distribution studies and in PK/PD breakpoint estimation studies. The present study illustrates the principles of using MCS to predict dosages of APR for the treatment of Salmonellosis in the piglets. The proposed dosage regimen is for Salmonellosis induced by *Salmonella* only. For other organisms, independent PK/PD studies will be required. 

CBP is used to determine whether the bacteria stain susceptibility or resistance. The CO_WT_, CO_PD_ and CO_CL_ should be considered for the determination of CBP [14]. Previous studies showed that there was a gap existing in CO_WT_ and CO_CL_. It was reported that CO_CL_ of danofloxacin against *Glaesserella parasuis* was 0.5 µg/mL, but the CO_WT_ of danofloxacin against *Glaesserella parasuis* was 8 µg/mL [28]. In our result, the CO_CL_ of APR against *Salmonella* was 8 µg/mL, but the CO_WT_ of APR against *Salmonella* was 32 µg/mL. So, our result was in accordance with the previous study. The possible reasons for the difference may be that the CO_WT_ establishment was due to the epidemiological characteristic of the selected bacteria affected by geographical differences of strains, susceptibility of strains, etc.

Our study investigated variations in the composition and function of ileum microbiota during the diarrheic piglet treatment process. It was consistent with the results of previous studies that *Firmicutes* was the dominant phylum in the ileum of piglet, and there were no significant differences in relative abundance among groups [14,18]. The change of the abundances of *Bacteroidetes* and *Euryarchaeota* was the most obvious during the evolution process (Figure 6A). At the genus level, *Methanobrevibacter*, *Prevotella*, *S24-7* and *Ruminococcaceae* were obtained as the highest abundance genus. The abundance of *Methanobrevibacter* increased significantly when APR treatment carried and decreased in cure and withdrawal period group. The abundance of *Prevotella* in test groups was significantly lower than that in the healthy group. A decreased of abundance in *S24-7* was observed after *Salmonella* infection and increased slightly after cure. *Ruminococcaceae* increased significantly after *Salmonella* infection and decreased significantly after APR treatment (Figure 6B). The results indicated that the *Salmonella* infection and APR treatment had a great impact on the ileum flora evolution. An increase of abundance in *Proteobacteria* was previously obtained as a biomarker for intestinal microbial community dysbiosis and a potential diagnostic criterion for disease [29]. Interestingly, in this study, *Proteobacteria* and *Lentisphaerae* were described as the biomarker of diarrheic piglets by LEfSe software (Figure 7B). This was consistent the previous study and another biomarker was found in our results. A previous study showed that *Prevotellacecea UCG-003* was a key node in diarrheic piglets upon co-correlation network analysis [30]. The genera of *Methanobrevibacter* and *Prevotella* was defined as the hub node by the Spearman network analysis; this result was different from previous study. The possible reason was that the sampling sites were different, and the composition of flora in ileum and piglet fecal were quite different. Another potential reason was the different effect of the selected challenge bacteria on the structure of flora. Further studies on the metabolites of the gut microbiome are needed to explore the correlation between these key genera and piglet diarrhea.

Antibiotics exert an effect on the amount and diversity of microbes causing change in functional diversity and colonization resistance against invading pathogens [31]. Previous study demonstrated that membrane transport, carbohydrate metabolism, amino acid metabolism, replication and repair, translation and energy metabolism were observed as the most abundant functions pathways by the prediction of microbial gene functions [32,33]. In this study, PICRUSt prediction revealed that the relative abundance of amino acid metabolism, carbohydrate metabolism, metabolism of cofactors and vitamins, metabolism of other amino acids, biosynthesis of other secondary metabolites, global and overview maps, replication and repair, glycan biosynthesis and metabolism, and lipid metabolism and energy metabolism. It was obvious that amino acid metabolism, replication and repair, and energy metabolism were obtained both in previous and in our results, so these KEGG pathways might play a key role in ileum flora evolution during the clinical treatment process.

## 4. Materials and Methods

### 4.1. Chemicals 

APR sulfate Standard (purity of 98%) was purchased from National Institutes for Food and Drug Control (Beijing, China). APR bulk (purity of 87.5%) was provided by the Institute of Veterinary Pharmaceuticals (HZAU, Wuhan, China). 

### 4.2. Salmonella Isolates

A total of 185 swine *Salmonella* strains were obtained from National Reference Laboratory of Veterinary Drug Residues (HZAU, Wuhan, China) and all the isolates were confirmed as the previous literature described [34]. 

### 4.3. Animals

Fifty-one four-week-old healthy crossbred (Duroc × Large × white × Landrace) pigs weighing 15 *±* 1 kg were purchased from Huazhong Agricultural University pig breeding farm. Eighty-one 7-week-old healthy female Kunming mice weighing 25 *±* 5 g were purchased from the Experimental Animal Center of Wuhan University. Prior to experiments, piglets and mice were raised for 7 days to acclimatize. All the animal experiments were approved by the Animal Ethics Committee of Huazhong Agricultural University (hzauch 2014-003) and the Animal Care Center, Hubei Science and Technology Agency in China (SYXK2013-0044). All animal experiments were conducted according to the committee guidelines for the Laboratory Animal Use and Care Committee in Hubei Science and Technology Agency. All efforts were used to reduce the pain and adverse effect of the animals.

### 4.4. Establishment of CO_WT_

The susceptibility of 185 swine *Salmonella* strains to APR was determined in triplicate by broth micro-dilution method according to Clinical and Laboratory Institute guidelines [35]. The MIC distribution was constructed as previously described [36]. The final ECOFF was measured as that MIC which captured at least 95% of the optimum MIC distributions calculated by the ECOFFinder software [37].

### 4.5. Model of Salmonella Infection

Thirteen isolates carrying more virulence genes was confirmed by multiplex Polymerase Chain Reaction (PCR); the primers and procedure are presented in Tables S9 and S10. Then, a model of *Salmonella* infection was established as follows. Briefly, female Kunming mice were infected at the age of 8 weeks. Food and water were provided ad libitum. Mice were inoculated by intraperitoneal infection with 200 μL of inoculum containing a total of 2 × 10^7^ or 2 × 10^9^ bacteria with three mice in each dose group. The control group was inoculated by intraperitoneal infection with 200 μL Luria-Bertan (LB) broth. The clinical symptoms of mice after *Salmonella* challenge were observed, and the target bacteria were isolated and identified after anatomical examination of poisoning mouse. Eventually, strains 3303, 10#, 34#, 10,643 and 5023, with an MIC of 4, 8, 16, 32 and 256 μg/mL, respectively, were selected for the further experiment. 

### 4.6. PD Study In Vitro and Ex Vivo

The MIC, MBC and MPC of APR against *Salmonella* 10# were determined in broth plasma and ileum fluid. PAE of APR against swine *Salmonella* was determined with the removal of drug methods. Strain 10# was exposed to 1 × MIC, 2 × MIC, 4 × MIC of APR. At 1 and 2 h, the removal drug culture was obtained by centrifuging and was re-cultured in fresh MH broth without APR. The colony-forming units (CFU) were determined at a series time-point. 

The in vitro and ex vivo killing curves of APR in broth and in ileum fluid were drawn by monitoring the CFU changes during the incubation of *Salmonella* 10# under a series concentration of APR (1/2 to 32 MIC) for a continuous time period (0, 1, 2, 4, 6, 8, 12 and 24 h) [28].

### 4.7. Samples Collection for PK Study

Twelve piglets were divided randomly into two groups with equal numbers of males and females in each group. Each group was treated via the intragastric route with a single dose of 30 mg/kg b.w. After administration, 2 mL blood samples were obtained at 0, 0.5, 1, 2, 3, 4, 6, 8, 10, 12, 24 and 48 h. Plasma was obtained followed by centrifugation at 3500 r/min for 10 min at 4 ℃. Ileum contents (5 g) from each pig were gently collected at time-point as that of the plasma. All the samples were stored at −80 °C prior to the analysis.

### 4.8. Samples Analysis for PK

The APR concentration in serum and ileum was determined using a high-performance liquid chromatography-tandem mass spectrometry (HPLC-MS/MS) method. In brief, serum samples (0.5 mL) and ileum contents (1 g) were treated with three times the volume of acetonitrile, vibrated for 30 s, and centrifuged at 14,000 r/min for 5 min. Then volume of 400 μL 4% H_3_PO_4_ was added to equal volume of supernatant to adjust the pH. An Oasis HLB 60-mg extraction cartridge (Waters) was successively conditioned with 3–5 mL of methanol before equilibrated with 3–5 mL of H_2_O. The cartridge was then rinsed with 0.8 mL of 5% methanol aqueous solution and dried under nitrogen for 4 min. Compounds of interest were eluted with 50 μL acetonitrile-methanol (50:25, *v*/*v*). The eluate was evaporated to dryness under a nitrogen stream at 30 °C, and the residue was taken up with 1 mL 0.1% formic acid in ammonium acetate-acetonitrile (15:85, *v*/*v*) before HPLC-MS-MS injection.

Ten microliters of each sample was injected onto a Shimadzu 30A system (Shimadzu, Japan), which included a binary pump, refrigerated autosampler (4 °C) and a temperature-controlled column compartment (30 °C). Chromatographic separation was performed using a Waters Acquity BEH C18 column (2.1 × 100 mm, 1.7 µm, Waters, Milford, MA, USA). The mobile phase consisted of A: 0.1% formic acid in ammonium acetate, and B: acetonitrile with a flow rate of 0.2 mL/min and a total run time of 4 min. A gradient of mobile phase B was utilized by decreasing the initial 85% linearly to 10% over 0.4 min, held there until 2.3 min, then returned to 85% by 2.4 min.

Detection of the APR was performed by an AB SCIEX QTRAP^®^ 5500 mass spectrometer (AB SCIEX, Framingham, MA, USA). The mass spectrometer was operated to monitor the monoisotopes of APR (m/z 540.3→378.4→344.4→217.4) using multiple reaction monitoring (MRM) under positive ionization mode. The ion spray voltage was set at 5500 V for positive monitoring, and the temperature was maintained at 550 °C. The nebulizing gas was high-purity nitrogen, and atomization gas was 65 psi, auxiliary gas was 60 psi and air curtain gas was 30 psi. Data processing was performed using the MultiQuant™ software (Version 3.0.3, AB SCIEX). The concentration–time data for APR in plasma and ileum samples harvested from healthy/infection piglets were analyzed by WinNonlin software (Version 6.3; Pharsight Inc., St. Louis, MO, USA) to obtain the pharmacokinetic parameters.

### 4.9. PK and PD Analyses, Dosage Prediction and Establishment of CO_PD_

To determine the PK/PD integration of APR in the ileum fluid, the AUC_0–24h_/MIC was calculated according to PK data and ex vivo PD data. Changes in bacterial abundance in ileum were integrated with the PK/PD parameters AUC_0–24h_/MIC using the sigmoid E_max_ model with WinNonlin as the previously described [27]. Based on the results of PK-PD modeling for the relationship between AUC_0–24h_/MIC and the ex vivo antibacterial effect, three levels of antibacterial effect of APR were quantified from the sigmoid E_max_ equation by determining AUC_0–24h_/MIC required for bacteriostatic action as previously described [38]. PK/PD parameter values corresponding to the E value (derived from the sigmoid E_max_ equation) in ileal fluid were used to deduce an optimal dose regimen [39]. The PD target was selected to calculate the probability of PTA. CO_PD_ was defined as the MIC at which the PTA was ≥90% under 10,000 trials with Crystal Ball software (version 7.2.2, Oracle, Austin, TX, USA) [39].

### 4.10. Infection Model and Clinical Trials

Thirty-nine healthy weaned piglets with body weight of (15 *±* 1 kg) were divided into seven groups including one blank control group (3 piglets), one positive control group infected without APR treatment (6 piglets) and five infected groups treated with APR (each 6 piglets), to obtain clinical data for APR in the treatment of *Salmonella* infection. Each piglet in the infected group was inoculated by intragastric administration with 50 mL of 1 × 10^10^ CFU/mL *Salmonella* strains 3303, 10#, 34#, 10,643 and 5023, two times per day and per piglet for three consecutive days. The blank control group was inoculated with blank LB broth. The dosage regimens were recommended by the PK-PD therapeutic dosage regimen. After challenged, these pigs were monitored daily for 4 days. 

### 4.11. Statistical Analysis for Establishment of CO_CL_

Simply, the data was firstly analyzed by the “WindoW” approach to obtain two parameters: “MaxDiff” (the method of maximum difference) and “CAR” (the curve (AUC) ratio) as the previously described [14]. According to the formula POC = 1/(1 + e^−a+bf (MIC)^) proposed by EUCAST, the obtained data in the “WindoW” approach was fitted by the nonlinear regression analysis using SPSS software. Then the MIC value corresponding to the cure rate of 90% was calculated. Eventually, CART software (version 6.0; Salford Systems, San Diego, CA, USA) was used for the establishment of CO_CL_ [14].

### 4.12. Samples Collection of Gut Flora 16S rRNA Gene Sequencing

Ileal contents samples were collected from the group infected by *Salmonella* strains 10# including five time nodes of before infection, infection, APR treatment, cure and withdrawal, sequentially named as the healthy group (GY), the infection group (GY0), the treatment group (GY24), the cure group (GY72) and the withdrawal period group (GY96). Except for 3 samples collected in the cure group, only 2 samples were collected at other times of sampling. Then content samples were snap-frozen in liquid nitrogen and stored at −80 °C. 

### 4.13. 16S rRNA Gene Sequencing

In short, microbial genomic DNA was extracted from 200 mg of ileum content samples using the TIANGEN DNA stool mini kit (TIANGEN, Beijing, China) following the manufacturer’s guidelines. Then, the *16S rRNA* gene of the V3-V4 region was amplified using specific primers as previously described [40]. The resulting PCR products were extracted from a 2% agarose gel and further purified using a TIANGEN gel DNA extraction kit (TIANGEN, Beijing, China). Purified PCR products were sequenced on an Illumina MiSeq platform by Bioacme Biotechnology Co., Ltd. (Shanghai, China). The raw data are available in the NCBI SRA database under BioProject accession number PRJNA761542.

### 4.14. Analysis of Microbial Diversity

To obtain high-quality clean tags, quality filtering of the raw tags was performed using the Quantitative Insights Into Microbial Ecology software (QIIME, version 1.9.1) quality-controlled process [41]. The chimaeric sequences were removed by using the UCHIME Algorithm and then the effective tags were obtained [42]. The sequences were clustered into OTUs at 97% similarity by UPARSE software (version 7.0.1001) [43]. In addition, the GreenGene database and MUSCLE software (version 3.8.31) were used to annotate the taxonomic information and conduct the multiple sequence alignment, respectively [44]. The alpha diversity index (Chao1, ACE, Shannon, Simpson indices), the beta diversity index, PCoA, NMDS and ANOSIM were conducted as previously described [45].

### 4.15. Microbial Taxonomic Analysis

To identify the genomic features of taxa differing in abundance between two or more biological conditions or classes, the LEfSe algorithm was used with the online interface Galaxy (http://huttenhower.sph.harvard.edu/lefse/, accessed on 4 September 2021) [46]. A size-effect threshold of 4.0 on the logarithmic LDA score was used for discriminative functional biomarkers. A Phylogenetic Investigation of Communities by Reconstruction of Unobserved States (PICRUSt, version 1.0.0) was performed to predict the ileal microbial community shift of the five groups of piglets. According to the calculation method described in the previous study [47], co-correlation networks were generated using the MENA (http://ieg4.rccc.ou.edu/MENA/, accessed on 1 September 2021) and the OTUs as target nodes, with edges (e.g., connecting nodes) representing significant negative (green) or positive (red) Spearman’s correlations. We retained OTUs when they had a Spearman’s correlation coefficient >0.5. The predicted genes and their function were aligned to KEGG database and the differences among groups were compared through software STAMP (version 2.1.3) [48].

## 5. Conclusions

This study firstly established the CO_WT_, CO_PD_ in ileum and CO_CL_ of APR against swine *Salmonella*, the value of these cutoffs were 32 µg/mL, 32 µg/mL and 8 µg/mL, respectively. According to the guiding principle of the CLSI, the final CBP in ileum was 32 µg/mL. Our results revealed the main evolution route in the composition of ileum microbiota of diarrheic piglets treated by APR. The change of the abundances of *Bacteroidetes* and *Euryarchaeota* was the most obvious during the evolution process. *Methanobrevibacter*, *Prevotella*, *S24-7* and *Ruminococcaceae* were obtained as the highest abundance genus. The abundance of *Methanobrevibacter* increased significantly when APR treatment carried and decreased in the cure and withdrawal period groups. The abundance of *Prevotella* in test groups was significantly lower than that in the healthy group. A decreased of abundance in *S24-7* was observed after *Salmonella* infection and increased slightly after cure. *Ruminococcaceae* increased significantly after *Salmonella* infection and decreased significantly after APR treatment. In addition, the genera of *Methanobrevibacter* and *Prevotella* were defined as the key node. Valine, leucine and isoleucine biosynthesis, D-Glutamine and D-glutamate metabolism, D-Alanine metabolism, peptidoglycan biosynthesis and biosynthesis of amino acids were the top five KEGG pathways in the ileum microbiota of piglets during the *Salmonella* infection and APR treatment process. 

## Figures and Tables

**Table 1:** Integrated PK parameters for APR in plasma and ileum contents after oral administration of 30 mg/kg b.w.

Parameter	Units	Plasma		Ileum Contents	
		Healthy Group	Infected Group	Healthy Group	Infected Group
C_max_	μg/mL	0.26 ± 0.01	0.24 ± 0.01	863.4 ± 18.2	750.5 ± 24.82
AUC_0–24h_	h·μg/mL	4.12 ± 0.76	3.41 ± 0.48	3287.25 ± 23.41	2855.83 ± 19.81
AUMC_0–24h_	h^2^·μg/mL	63.68 ± 2.12	45.13 ± 1.71	19,965.83 ± 151.2	17,421.25 ± 132.5
T_max_	H	4.0 ± 0.0	4.0 ± 0.0	3.0 ± 0.0	3.0 ± 0.0
T_1/2_	H	20.00 ± 1.02	15.77 ± 1.93	8.74 ± 1.01	7.83 ± 0.78
MRT_0–24h_	H	15.47 ± 0.92	13.22 ± 1.02	6.07 ± 0.24	6.10 ± 0.42
CL/F	mL/h/kg	5.69 ± 0.01	7.76 ± 0.02	9.0 ± 0.02	10.04 ± 0.01

Note: C_max_ = maximum concentration, AUC_0–24h_ = area under concentration curve, AUMC_0–24h_ = first-order area under concentration curve, T_1/2λ_ = elimination half-life, T_max_ = time of maximum concentration, MRT_0–24h_ = mean residence time, CL/F = body clearance scaled by bioavailability.

**Table 2:** Ex vivo PK parameters of APR against swine *Salmonella*.

Parameter	Unit	Value	
		Healthy Group	Infected Group
E_max_	Log10 CFU/mL	4.25	3.48
E_0_	h	−5.69	−5.33
EC_50_	Log10 CFU/mL	47.16	49.37
N	-	27.25	6.10
E_max_-E_0_	Log10 CFU/mL	9.94	8.81
AUC_0–24h_/MIC (E = 0)	h	46.65	46
AUC_0–24h_/MIC (E = −3)	h	48.90	58.38
AUC_0–24h_/MIC (E = −4)	h	49.98	65.53

## Data Availability

The sequencing data was submitted to the National Center for Biotechnology Information Sequence Read Archive (SRA) under Accession No. PRJNA761542.

## Supplementary Materials

The following are available online at https://www.mdpi.com/article/10.3390/ijms23031424/s1.
