# Supplementary material for: Clinical Breakpoint of Apramycin to Swine Salmonella and Its Effect on Ileum Flora
Source: Int J Mol Sci. 2022 Jan 26;23(3):1424. doi: 10.3390/ijms23031424 (PMC8835974; doi:10.3390/ijms23031424)
Supplement: Supplementary file 1 [file ijms-23-01424-s001.zip › ijms-1482036-supplementary.pdf]

Supplementary data

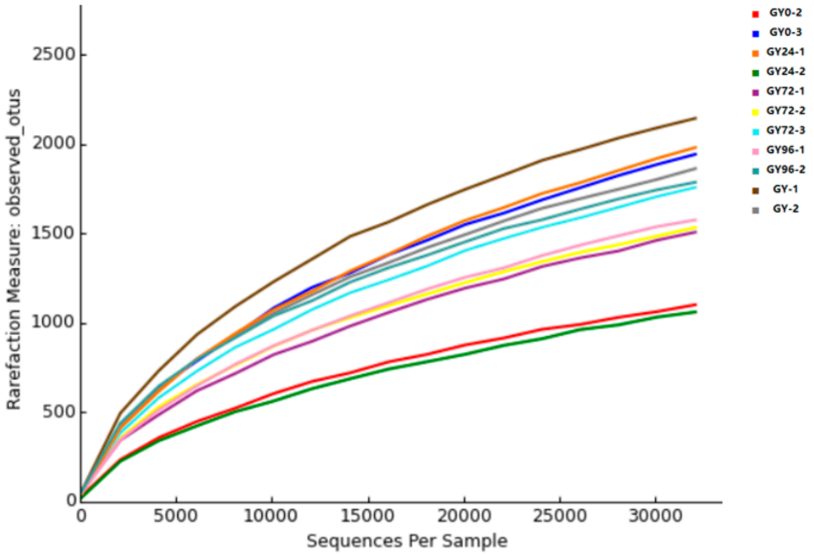

Figure S1. Rarefaction curves of the OTU number in the ileal contents samples.

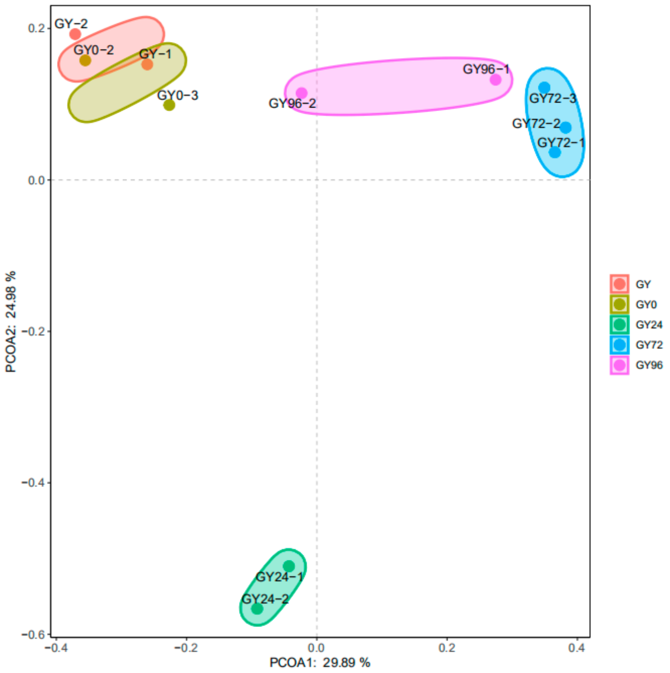

Figure S2. Principal coordinate analysis (PCoA) shows the ileal microbiota of the healthy, infection, treatment, cure and withdraw period group.

Table S1. Statistics of antibacterial activity of APR against swine *Salmonella.*

MIC(μg/mL)	NO. strain						Total
	4759	1880	279	8515	7897	6604	
	7900	8515	9288	9862	277-1	9105	
4	8519	6#	3303	10016	250j	10234	22
	9727	9530	10454	9#			
	8414	7963	1957	9727	6602	1959	
	8515	7897	23#	9505	6604	9104	
	2088	1939	8287	246j	10315	9899	
	9504	4433	9503	252-1	8771	251	
	9862	7968	10025	7900	9105	9727	
	7904	10455	9790	9387	293	1#	
	10#	10314	1959	9530	10#	9434	
	8997	3#	7964	293	9286	4776	
	7903	9103	9848	8447	12#	349	
	7904	9504	4447	8091	7897	253j	
	9227	295	277-2	8#	10025	3297	
	10318	4414	10234	467	3801	2481	
8	3#	6601	9103	6#	10316	210-1	141
	10643	1957	3471	9434	2087	1822a	
	8447	9508	9287	9286	2891	6220	
	C77-31	C78-4	6883	2001	2883	2116	
	8398	2932	6410	7227	8913	11#	
	3827	9289	8997	10312	9#	4782	
	7913	12#	0596	215-1	9530	9643	
	9849	7204	8019	8518	10316	2932	
	4444	4435	284	8400	6433	2372	
	7889	6380	8518	6605	5062	277-2	
	6433	9599	8772	9436	4439	8#	
	251	7892	6391				
16	10642	1990	34#				3
32	10642	279	283				3
128	0596						1
	8777	5023	8913	251	5023	5062	
>256	6220	10024	7227	2814	4776	8001	13
	7904						

Table S2. The PAE of APR against swine *Salmonella*.

APR concentration (μg/mL)	PAE(h)	
	Exposure 1h	Exposure 2h
1×MIC	0.51	0.45
2×MIC	0.66	1.25
4×MIC	1.42	6

Table S3. Concentrations of APR in swine plasma after a single dose administration.

Time(h)	Concentration (μg/mL)	
	Healthy group	Infection group
0.5	0.08±0.02	0.13±0.01
1	0.23±0.01	0.21±0.01
2	0.23±0.03	0.22±0.02
4	0.26±0.03	0.24±0.03
6	0.18±0.01	0.17±0.02
8	0.14±0.04	0.12±0.02
10	0.11±0.02	0.09±0.01
12	0.05±0.01	0.04±0.01
24	0.04±0.01	0.02±0.02
48	-	-

Note: The concentrations of APR was lower than the limit of quantitation of the method.

Table S4. Concentrations of APR in swine ileum fluid after a single dose administration.

Time(h)	Concentration (μg/mL)	
	Healthy group	Infection group
0.5	3.5±0.57	2.7±0.28
1	21.8±1.31	20.1±1.23
2	356.1±21.15	259.6±19.32
3	863.4±20.22	750.5±18.98
4	566.5±18.92	470.6±20.33
6	263.3±8.9	260.9±15.32
8	91.8±4.32	84.7±9.32
10	70.4±11.2	64.7±14.2
12	25.5±9.1	20.2±3.22
24	7.2±2.3	7.9±1.21
48	3.4±0.9	1.6±0.32

Table S5. PTA of APR against swine *Salmonella* at different MICs.

MIC(μg/mL)	PTA(%)
8	100
16	100
32	100
48	90
64	0

Table S6. Probablity of cure (POC) of APR against swine *Salmonella*.

Group	MIC	Total	Mortality	Mortality rate	Effective rate	POC
Blank	-	6	0	0	-	-
Infection	8 μg/mL	6	2	33.33%	-	-
	4 μg/mL	6	0	0	100%	100%
	8 μg/mL	6	0	0	100%	100%
Treatment	16 μg/mL	6	1	16.67%	83.33%	83.33%
	32 μg/mL	6	1	16.67%	83.33%	66.67%
	256 μg/mL	6	2	33.33%	66.67%	66.67%

Table S7. Statistics of KEGG pathway at level 2 and the total sequences contained in each sample.

Sample ID	Number of seqs	Number of OUTs
GY-1	32867	2165
GY-2	46923	2171
GY0-2	74959	1570
GY0-3	49914	2345
GY24-1	62300	2638
GY24-2	80035	1675
GY72-1	43435	1748
GY72-2	48936	1828
GY72-3	52285	2151
GY96-1	42356	1775
GY96-2	35374	1862

Table S8. Statistics of predicted KEGG pathway at level 2.

Taxonomy	GY	GY0	GY24	GY72	GY96
Amino acid metabolism	103685.67	162575.55	181753.30	132447.10	110417.07
Carbohydrate metabolism	94926.63	151329.46	153340.79	118311.85	103352.12
Metabolism of cofactors and vitamins	91562.96	129904.87	156523.41	110035.48	90771.68
Metabolism of other amino acids	64371.93	97016.16	104714.22	79498.50	68982.66
Biosynthesis of other secondary	56935.88	89101.25	99384.26	74338.36	62024.94

metabolites					
Global and overview maps	56649.88	88656.09	101255.76	71141.48	59469.47
Replication and repair	52942.87	84145.45	92841.42	64031.13	54996.90
Glycan biosynthesis and metabolism	42955.01	59068.49	56796.53	48997.44	42309.45
Lipid metabolism	42900.59	64979.65	70279.29	53460.16	45099.32
Energy metabolism	42817.46	66812.19	72976.28	53356.44	45185.59
Translation	31056.01	49081.10	54388.67	38257.99	30912.96
Folding, sorting and degradation	28255.08	44572.40	54531.45	35700.37	29482.04
Xenobiotics biodegradation and metabolism	26359.12	35974.79	39094.59	34334.58	30907.46
Metabolism of terpenoids and polyketides	26186.11	41273.22	39939.94	31980.04	27281.01
Cell motility	23080.22	50208.25	27069.81	25009.78	23777.55
Drug resistance: antimicrobial	20364.23	32558.71	34149.08	24379.87	21217.97
Cell growth and death	19257.36	29396.20	32477.47	23731.00	20218.96
Nucleotide metabolism	15858.81	24395.62	26759.13	19172.69	15987.62
Membrane transport	14837.85	24553.73	23538.08	18750.94	17022.08
Cellular community - prokaryotes	12847.13	20559.25	20661.76	15469.64	12965.96
Endocrine system	8857.73	12834.73	15283.71	10647.59	8832.28
Drug resistance: antineoplastic	8273.63	12750.24	13167.54	9577.59	8291.52
Signal transduction	8145.05	12747.69	12644.18	9707.46	8596.66
Cancer: overview	5150.56	8189.85	8318.22	6236.32	5564.74
Infectious disease: bacterial	4992.47	7349.36	7032.68	5846.99	5050.03
Aging	4786.04	6777.10	6521.97	4999.96	4393.88
Endocrine and metabolic disease	4312.13	6771.01	7745.54	5410.71	4386.06
Transcription	3095.10	4791.62	5509.72	4072.85	3136.03
Transport and catabolism	2850.55	3503.01	4059.20	3261.90	2732.52
Immune system	2732.40	4557.95	5179.47	3276.91	2799.25
Nervous system	2450.44	4335.19	5076.74	3141.24	2811.08

Environmental adaptation	1734.72	2847.27	2864.20	2023.44	1750.07
Cardiovascular disease	1644.54	2512.49	3084.11	2206.41	1844.49
Digestive system	1456.64	1131.12	858.00	1349.99	977.72
Chemical structure transformation maps	1366.00	560.50	1230.00	3964.67	4233.00
Neurodegenerative disease	1199.29	1914.59	1160.65	1279.58	1312.53
Excretory system	1132.61	1436.00	1321.68	996.64	1115.54
Immune disease	1109.01	1737.41	943.44	1444.78	1080.89
Cancer: specific types	860.74	1192.18	1202.98	1078.22	983.50
Infectious disease: parasitic	383.48	265.30	237.13	540.28	478.75
Infectious disease: viral	219.56	329.67	338.02	440.93	299.46
Substance dependence	59.23	76.55	44.18	146.27	136.18

Table S9. Primer used for identification of virulence genes of *Salmonella*.

Gene Name	Premers	Sequences(5′-3′)	Sizes（bp)
spvB	*spvB*-F	GCTGTTCACCCTAGTGTTTGAC	811
	spvB-R	GCTGTTCACCCTAGTGTTTGAC	
spvR	*spvR*-F	CGCTACGCCTTTGATGTGC	337
	spvR-R	TCGGGAATCGCTTGTCTGC	
avrA	*avrA*-F	CCTGTATTGTTGAGCGTCTGG	425
	avrA-R	AGAAGAGCTTCGTTGAATGTCC	
ssaQ	*ssaQ*-F	GGGGGAATCTACGCAAGGG	235
	ssaQ-R	CACCTACAGGCAAAACGTCC	
sopE1	*sopE1*-F	ACTCCTTGCACAACCAAATGCGGA	527
	sopE1-R	CTGTCTCTGCATTTCGCCACC	
fljB	*fljB*-F	ATGGCACAAGTCATTAATACAAAC	1515
	fljB-R	ACGCAGTAAAGAGAGGAC	

Table S10. Volume of reagent for multiple PCR（μL）.

Components	2×Taq Master Mix	Template	F-premer	R-premer	ddH_2O	Total
Volume	12.5	2	1	1	8.5	25

Note: The procedure was pre denaturation at 95 ℃ for 3min, denaturation at 95 ℃ for 15s, annealing at 55 ℃ for 15s, extension at 72 ℃ for 30s, amplification for 30 cycles, and final extension at 72 ℃ for 5min.
